# Supplementary material for: Elementary 3D organization of active and silenced E. coli genome
Source: Nature. 2025 Aug 13;645(8082):1060–70. doi: 10.1038/s41586-025-09396-y (PMC12460168; doi:10.1038/s41586-025-09396-y)
Supplement: Supplementary file 1 — This file contains Supplementary Fig. 1 and Supplementary Table legends. [file 41586_2025_9396_MOESM1_ESM.pdf]

nature portfolio

https://doi.org/10.1038/s41586-025-09396-y

Supplementary information

Elementary 3D organization of active and silenced *E. coli* genome

In the format provided by the authors and unedited

Elementary 3D organization of active and silenced *E. coli* genome

Alexey A. Gavrilov[1,2#], Ilya Shamovsky[2#], Irina Zhegalova[1§], Sergey Proshkin[3], Yosef Shamovsky[2], Grigory Evko[1], Vitaly Epshtein[2], Aviram Rasouly[2], Anna Blavatnik[2], Sudipta Lahiri[2], Eli Rothenberg[2], Sergey V. Razin[1,4*], and Evgeny Nudler[2,5*]

[1]Institute of Gene Biology, Russian Academy of Sciences, Moscow 119334, Russia

[2]Department of Biochemistry and Molecular Pharmacology, New York University Grossman School of Medicine; New York, NY 10016, USA

[3]Engelhardt Institute of Molecular Biology, Russian Academy of Sciences, Moscow 119991, Russia

[4]Department of Molecular Biology, Faculty of Biology, M.V. Lomonosov Moscow State University, 119991 Moscow, Russia

[5]Howard Hughes Medical Institute, NYU Langone Health; New York, NY 10016, USA

[#]Equal contribution

[§]Present affiliation: Institute for Medical Engineering and Science, Massachusetts Institute of Technology, Cambridge, MA 02139, USA

*Correspondence: evgeny.nudler@nyulangone.org; sergey.v.razin@gmail.com

Table of Content

Supplementary Fig. 1.	Page 3
Supplementary Table 1 Legend	Page 4
Supplementary Table 2 Legend	Page 4
Supplementary Table 3 Legend	Page 4
Supplementary Table 4 Legend	Page 4
Supplementary Table 5 Legend	Page 4
Supplementary Table 6 Legend	Page 4
Supplementary Table 7 Legend	Page 4
Supplementary Table 8 Legend	Page 4
Supplementary Table 9 Legend	Page 4

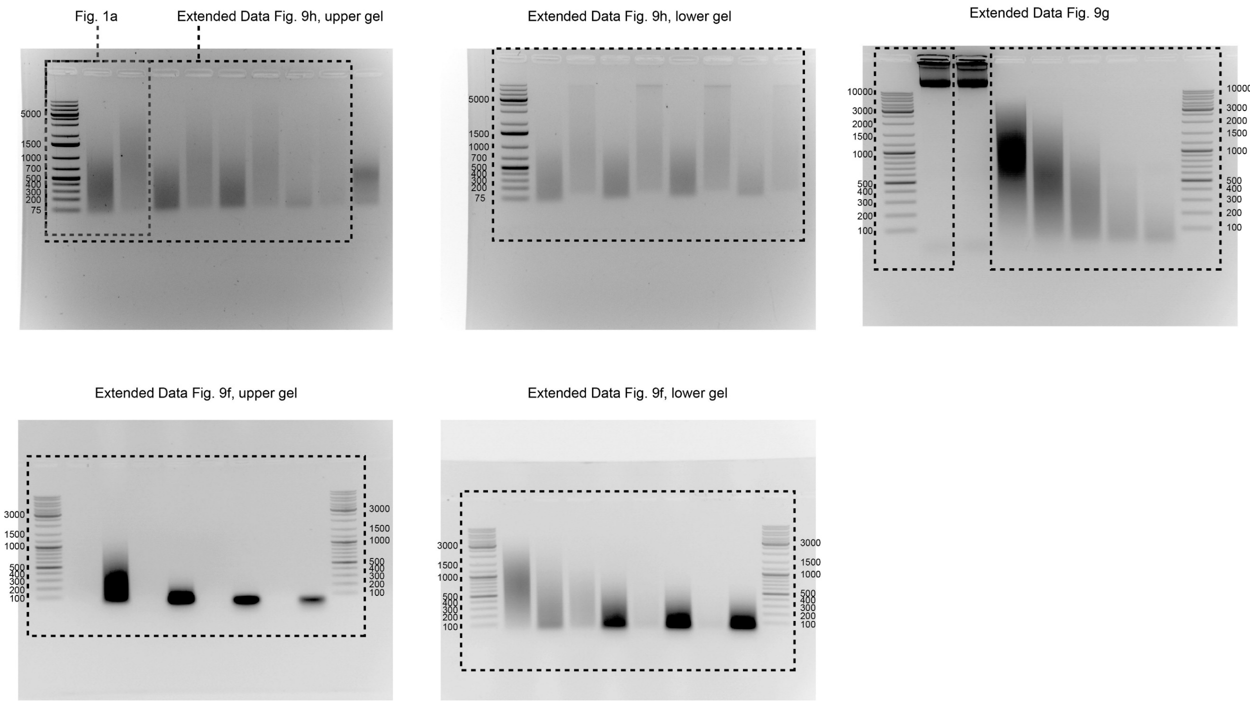

Supplementary Fig. 1. Uncropped gel images. Cropped regions shown in the main text are those indicated within dashed squares.

Supplementary Table 1. Changes in gene expression in response to H-NS and StpA knockouts, or exposure of *E. coli* cells to 200 µg/mL netropsin, as determined by RNA-seq. The two rightmost columns indicate whether a gene is classified as an HTG and whether it overlaps with CHIN(s) in wild-type *E. coli* cells.

Supplementary Table 2. Statistics of Micro-C read mapping and filtering.

Supplementary Table 3. Coordinates of operons and their transcriptional levels in wild-type *E. coli* cells under normal and heat shock conditions as determined by Red-C. The two rightmost columns show the frequencies of promoter-terminator and intra-operon contacts in wild-type *E. coli* cells.

Supplementary Table 4. Coordinates of OPCIDs and their transcriptional levels in wild-type *E. coli* cells under normal and heat shock conditions as determined by Red-C.

Supplementary Table 5. Coordinates of CHINs and their transcriptional levels in wild-type *E. coli* cells under normal and heat shock conditions as determined by Red-C. The rightmost column shows CHIN status in Δ*hns* cells.

Supplementary Table 6. Coordinates of CHIDs and their transcriptional levels in wild-type *E. coli* cells under normal and heat shock conditions as determined by Red-C.

Supplementary Table 7. Coordinates of CHIN contacts.

Supplementary Table 8. Coordinates of CHINs annotated by Chromosight program.

Supplementary Table 9. Sequences of the dual sgRNA cassettes used in PLA experiments.
