# Supplementary material for: Elementary 3D organization of active and silenced E. coli genome
Source: Nature. 2025 Aug 13;645(8082):1060–70. doi: 10.1038/s41586-025-09396-y (PMC12460168; doi:10.1038/s41586-025-09396-y)
Supplement: Supplementary file 2 — Reporting Summary [file 41586_2025_9396_MOESM2_ESM.pdf]

nature portfolio

Corresponding author(s): Evgeny Nudler, Sergey Razin

Last updated by author(s): Jun 24, 2025

Reporting Summary

Nature Portfolio wishes to improve the reproducibility of the work that we publish. This form provides structure for consistency and transparency in reporting. For further information on Nature Portfolio policies, see our Editorial Policies and the Editorial Policy Checklist.

Statistics

For all statistical analyses, confirm that the following items are present in the figure legend, table legend, main text, or Methods section.

n/a	Confirmed	
☐	☒	The exact sample size (*n*) for each experimental group/condition, given as a discrete number and unit of measurement
☐	☒	A statement on whether measurements were taken from distinct samples or whether the same sample was measured repeatedly
☐	☒	The statistical test(s) used AND whether they are one- or two-sided<br>*Only common tests should be described solely by name; describe more complex techniques in the Methods section.*
☐	☒	A description of all covariates tested
☐	☒	A description of any assumptions or corrections, such as tests of normality and adjustment for multiple comparisons
☐	☒	A full description of the statistical parameters including central tendency (e.g. means) or other basic estimates (e.g. regression coefficient) AND variation (e.g. standard deviation) or associated estimates of uncertainty (e.g. confidence intervals)
☐	☒	For null hypothesis testing, the test statistic (e.g. *F*, *t*, *r*) with confidence intervals, effect sizes, degrees of freedom and *P* value noted<br>*Give P values as exact values whenever suitable.*
☒	☐	For Bayesian analysis, information on the choice of priors and Markov chain Monte Carlo settings
☒	☐	For hierarchical and complex designs, identification of the appropriate level for tests and full reporting of outcomes
☐	☒	Estimates of effect sizes (e.g. Cohen's *d*, Pearson's *r*), indicating how they were calculated

Our web collection on statistics for biologists contains articles on many of the points above.

Software and code

Policy information about availability of computer code

Data collection	No custom software was used.
Data analysis	Micro-C and Hi-C reads were processed using distiller-nf version 0.3.4. Mapping was performed using BWA. The sample correlation was computed using HiCRep. Scalings were calculated using cooltools v. 0.7.0. Average contact maps were plotted using coolpuppy v1.1.0 and cooltools v0.5.4. Piles-ups were generated using the custom function plot_around_loop with some imports from pybbi package v0.4.0. The motif analysis was performed using HOMER v4.11.1 and tomtom version 5.5.5 from the MEME suite. RNA-seq data processing was conducted using the nf-core/rnaseq v3.16.0 workflow. Differential gene expression analysis was performed with the DESeq2 package. Red-C sequencing reads were processed using the RedClib computational pipeline (https://github.com/agalitsyna/RedClib).

For manuscripts utilizing custom algorithms or software that are central to the research but not yet described in published literature, software must be made available to editors and reviewers. We strongly encourage code deposition in a community repository (e.g. GitHub). See the Nature Portfolio guidelines for submitting code & software for further information.

Data

Policy information about availability of data

All manuscripts must include a data availability statement. This statement should provide the following information, where applicable:

- Accession codes, unique identifiers, or web links for publicly available datasets
- A description of any restrictions on data availability
- For clinical datasets or third party data, please ensure that the statement adheres to our policy

Micro-C and Hi-C fastq raw reads and cool contact matrices are available under GEO accession: GSE272161.
Red-C fastq raw reads and tsv files with contacts are available under GEO accession: GSE272161.
RNA-seq raw reads are available under GEO accession: GSE272161.

Research involving human participants, their data, or biological material

Policy information about studies with human participants or human data. See also policy information about sex, gender (identity/presentation), and sexual orientation and race, ethnicity and racism.

Reporting on sex and gender	Not applicable to this study.
Reporting on race, ethnicity, or other socially relevant groupings	Not applicable to this study.
Population characteristics	Not applicable to this study.
Recruitment	Not applicable to this study.
Ethics oversight	Not applicable to this study.

Note that full information on the approval of the study protocol must also be provided in the manuscript.

Field-specific reporting

Please select the one below that is the best fit for your research. If you are not sure, read the appropriate sections before making your selection.

☒ Life sciences ☐ Behavioural & social sciences ☐ Ecological, evolutionary & environmental sciences

For a reference copy of the document with all sections, see nature.com/documents/nr-reporting-summary-flat.pdf

Life sciences study design

All studies must disclose on these points even when the disclosure is negative.

Sample size	Sample sizes were based on standard practice in similar studies and were deemed sufficient to ensure reproducibility and detect consistent effects across independent replicates.
Data exclusions	No data was excluded.
Replication	Micro-C experiments were performed in two biological replicates except the experiments without adding DNA ligase and with varying cultivation/fixation temperature and experiments with antibiotics ciprofloxacin, novobiocin and netropsin, which were performed in one repeat. Hi-C and Red-C experiments were performed in two biological replicates. RT-qPCR experiments were performed in at least four biological replicates. RNA-seq experiments were performed in five biological replicates. TEM and PLA experiments were performed in two repeats. All experiments showed high reproducibility between replicates.
Randomization	Randomization was not applicable, as the study involved controlled experiments using cell cultures with uniformly applied conditions, eliminating the need for random allocation.
Blinding	Blinding was not required in this study, as it did not involve a clinical trial with human participants.

Reporting for specific materials, systems and methods

We require information from authors about some types of materials, experimental systems and methods used in many studies. Here, indicate whether each material, system or method listed is relevant to your study. If you are not sure if a list item applies to your research, read the appropriate section before selecting a response.

Materials & experimental systems

n/a	Involved in the study
☒	☐ Antibodies
☒	☐ Eukaryotic cell lines
☒	☐ Palaeontology and archaeology
☒	☐ Animals and other organisms
☒	☐ Clinical data
☒	☐ Dual use research of concern
☒	☐ Plants

Methods

n/a	Involved in the study
☒	☐ ChIP-seq
☒	☐ Flow cytometry
☒	☐ MRI-based neuroimaging

Plants

Seed stocks	*Report on the source of all seed stocks or other plant material used. If applicable, state the seed stock centre and catalogue number. If plant specimens were collected from the field, describe the collection location, date and sampling procedures.*
Novel plant genotypes	*Describe the methods by which all novel plant genotypes were produced. This includes those generated by transgenic approaches, gene editing, chemical/radiation-based mutagenesis and hybridization. For transgenic lines, describe the transformation method, the number of independent lines analyzed and the generation upon which experiments were performed. For gene-edited lines, describe the editor used, the endogenous sequence targeted for editing, the targeting guide RNA sequence (if applicable) and how the editor was applied.*
Authentication	*Describe any authentication procedures for each seed stock used or novel genotype generated. Describe any experiments used to assess the effect of a mutation and, where applicable, how potential secondary effects (e.g. second site T-DNA insertions, mosiacism, off-target gene editing) were examined.*
